# Micromorphology, Ultrastructure and Histochemistry of *Commelina benghalensis* L. Leaves and Stems

**DOI:** 10.3390/plants10030512

**Published:** 2021-03-09

**Authors:** Kareshma Doolabh, Yougasphree Naidoo, Yaser Hassan Dewir, Nasser Al-Suhaibani

**Affiliations:** 1 School of Life Sciences, University of KwaZulu-Natal, Westville, Private Bag X54001, Durban 4000, South Africa; 210504977@stu.ukzn.ac.za (K.D.); naidooy1@ukzn.ac.za (Y.N.); 2 Plant Production Department, PO Box 2460, College of Food and Agriculture Sciences, King Saud University, Riyadh 11451, Saudi Arabia; nsuhaib@ksu.edu.sa; 3 Department of Horticulture, Faculty of Agriculture, Kafrelsheikh University, Kafr El-Sheikh 33516, Egypt

**Keywords:** alkaloids, non-glandular trichomes, hooked, microscopy, morphology, multicellular, phenols

## Abstract

*Commelina benghalensis* L. is used as a traditional medicine in treating numerous ailments and diseases such as infertility in women, conjunctivitis, gonorrhea, and jaundice. This study used light and electron microscopy coupled with histochemistry to investigate the micromorphology, ultrastructure and histochemical properties of *C. benghalensis* leaves and stems. Stereo and scanning electron microscopy revealed dense non-glandular trichomes on the leaves and stems and trichome density was greater in emergent leaves than in the young and mature. Three morphologically different non-glandular trichomes were observed including simple multicellular, simple bicellular and simple multicellular hooked. The simple bicellular trichomes were less common than the multicellular and hooked. Transmission electron micrographs showed mitochondria, vesicles and vacuoles in the trichome. The leaf section contained chloroplasts with plastoglobuli and starch grains. Histochemical analysis revealed various pharmacologically important compounds such as phenols, alkaloids, proteins and polysaccharides. The micromorphological and ultrastructural investigations suggest that *Commelina benghalensis* L. is an economically important medicinal plant due to bioactive compounds present in the leaves and stems.

## 1. Introduction

*Commelina benghalensis* L. (Commelinaceae), also known as the Benghal dayflower, is a perennial herb native to the tropics of Africa and Asia [1]. The plant is widely distributed in the northern and eastern regions of South Africa [2]. This plant species has a low risk of extinction which could be attributed to its rapid weed-like growth [3,4] and is thus categorized as an invasive weed in various parts of the world [4]. The plants grow along roadsides and lawns, home gardens, crop fields, waste sites, agricultural sites and forest edges [5]. Globally, *C. benghalensis* is used as a traditional medicinal plant. In Africa, it is traditionally used to treat infertility in women, gonorrhea, conjunctivitis, malaria and jaundice [6,7,8]. Various studies have shown that the bioactive compounds in extracts of *C. benghalensis* possess anti-inflammatory, antimicrobial, antidiabetic, antidiarrheal and analgesic properties [1,9,10,11,12,13].

Plants can produce secondary metabolites which add to a plant’s medicinal value [14]. These metabolites are produced and stored in specialized tissues or organs situated internally or on the plant’s surfaces [15,16]. Trichomes are an example of such specialized plant tissue that originates from small protrusions on the epidermis of the reproductive and vegetative organs [17,18]. Trichomes are able to synthesize, store and secrete large volumes of a variety of metabolites [18]. These metabolites have commercial value where they are presented as fragrances, natural pesticides, pharmaceuticals and food additives [18]. The exploitation of bioactive constituents within trichomes is brought about due to their easy accessibility as plant surface appendages [18,19]. Classified as non-glandular (NGT) or glandular secreting (GST) [17], trichomes differ in their morphological and mechanical characteristics (size, orientation, shape, density, and surface texture) that affect a plant’s ecology and physiology [19]. The anatomical characteristics of trichomes make them one of the most useful tools in taxonomy [20]. The metabolites found in trichomes may act as a defense mechanism against pathogens and herbivores and show great potential in human medicine and nutrition [21]. In addition to the bioactive constituents in trichomes, calcium oxalate crystals can be found among several other cell contents that also exhibit a protective and defensive role in plants [22]. Despite the extensive ethnobotanical uses of *C. benghalensis*, there is a dearth of detailed scientific research on the micromorphology and ultrastructure of trichomes present on the leaves and stems of this plant species. Previous ultrastructural studies focused on the effect of herbicides on leaves [23], the coexistence of apoplastic and symplastic phloem loading on leaves [24] and the transfer of fluorescent dye symplastically through the leaf tissue [25]. There is no research identifying the ultrastructure of trichomes of *C. benghalensis* or the presence of secondary metabolites within these trichomes by means of histochemical staining. This study would be the first account illustrating the ultrastructural features of trichomes in *C. benghalensis*. The identification of micromorphological features would be beneficial due to its economic importance as a medicinal plant. The purpose of this descriptive study was to investigate the micromorphology and ultrastructure of trichomes on the leaves and stems of *C. benghalensis* and to identify the accessible sites of bioactive constituents, histologically, in order to aid further research on this specific species.

## 2. Materials and Methods

### 2.1. Plant Material Collection

*Commelina benghalensis* leaves and stems were collected at the University of Kwa-Zulu Natal, Westville campus (UKZN) in Durban. The species was identified using herbarium specimens and a voucher specimen (18259) was deposited in the UKZN Ward Herbarium, Westville Campus, Durban. For microscopic analyses, fresh leaves and stems were collected and prepared. Leaves were categorized into three developmental stages based on leaf length; emergent (±1.5−3 cm), young (±3−4 cm) or mature (±4 cm).

### 2.2. Stereomicroscopy

Trichome distribution on the adaxial and abaxial leaf surfaces was examined. Approximately 3–4 leaves per developmental stage were analyzed and imaged using a Nikon AZ100 stereomicroscope with Nikon Fiber Illuminator on the NIS-Elements Software, NIS-elements D 3.00 (Tokyo, Japan). The mid-vein regions of the leaves and the stem were also examined and imaged.

### 2.3. Scanning Electron Microscopy (SEM)

Fresh leaves (approx. 6) from each developmental stage were collected and freeze-dried. The leaves were trimmed into segments (4 mm × 4 mm) and quenched in subcooled liquid nitrogen. Leaf segments were freeze-dried using an Edwards–Modulyo freeze dryer for 72 h (−60 °C in a vacuum of 10–2 Torr). The segments were mounted and secured onto brass stubs using carbon conductive tape. The segments were coated in gold using a Polaron SC500 Sputter Coater and viewed on a LEO 1450 SEM at a working distance of 14–19 mm. Smart SEM version 5.03.06. was used for image capture.

### 2.4. Transmission Electron Microscopy (TEM)

Leaves (approx. 12) and stems were trimmed into segments and fixed in 2.5% glutaraldehyde for 24 h. The segments were washed three times for 5 min in a 0.1 M phosphate buffer (pH 7.2). Postfixation was achieved by immersing segments overnight in 0.5% osmium tetroxide. Segments were washed three times in phosphate buffer for 5 min. Segments were dehydrated by washing twice for 5 min in 30, 50, and 75% acetone, followed by two washes in 100% acetone for 10 min each. The segments were then washed twice in propylene for 10 min each. Infiltration was achieved by immersing sections in Spurr’s resin [26]: propylene oxide (25:75, 50:50, 75:25, and 100:0) for 18–24 h. The infiltrated segments were placed into a silicon mold containing whole resin and polymerized in an oven for 8 h at 65 °C. Glass knives were created using an LKB knife maker 7801A (LKB Bromma, Bromma, Sweden). These knives were used to obtain semi-thin (survey) sections cut from the resin blocks containing the leaf and stem segments on the Reichert−Jung Ultra−microtome. Sections were stained with 1% Toluidine Blue and viewed on a Nikon Eclipse 80i compound light microscope (Nikon, Tokyo, Japan) with the NIS-Elements imaging software package. Ultrathin sections were cut at 100 nm using a Reichert−Jung Ultracut−E ultra−microtome (Leica Microsystems, Wetzlar, Germany) and picked onto copper grids. Sections were stained using 2.5% uranyl acetate and lead citrate. The sections were viewed on a JEOL 2100 High−Resolution TEM (Tokyo, Japan) at 200 KeV.

### 2.5. Histochemistry

Histochemical analyses were conducted on fresh leaf and stem sections according to standard staining procedures. Stains used were 0.1% Ruthenium red [27]; 1% Toluidine Blue [28,29]; Methylene blue [30]; 0.01% Calcofluor white [31]; Fast green [32]; 0.25% Coomassie blue for proteins [33]; Sudan Black B [34] 10% Ferric chloride [27,35]; under UV light [36]; Safranin [37,38]; Wagner’s and Dittmar reagent [39] and 0.01% Acridine orange [40].

Approximately 10 leaves and stem tissue were sectioned with an Oxford Vibratome into 150–200 μm thick sections, stained and viewed using a Nikon Eclipse 80i compound light microscope (Tokyo, Japan). Images were captured with the NIS-Elements imaging software package. Fluorescence microscopy was carried out using Nikon Eclipse 80i compound light microscope (Tokyo, Japan) equipped with a Nikon DS-Fil fluorescent camera with excitation and DM wavelengths of 330 nm and 400 nm, respectively.

## 3. Results and Discussion

### 3.1. Stereomicroscopy of Leaves and Stem of C. benghalensis

**Figure 1:**
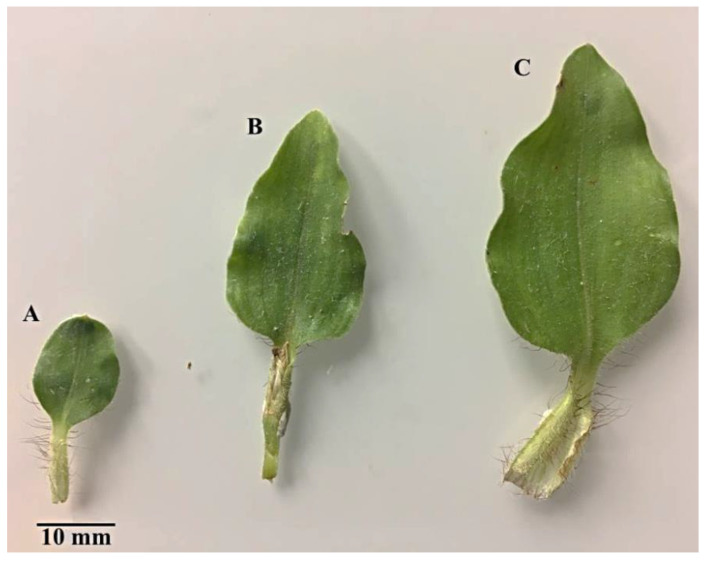
*C. benghalensis* leaves at different developmental stages. (**A**) Emergent; (**B**) Young; (**C**) Mature leaf.

**Figure 2:**
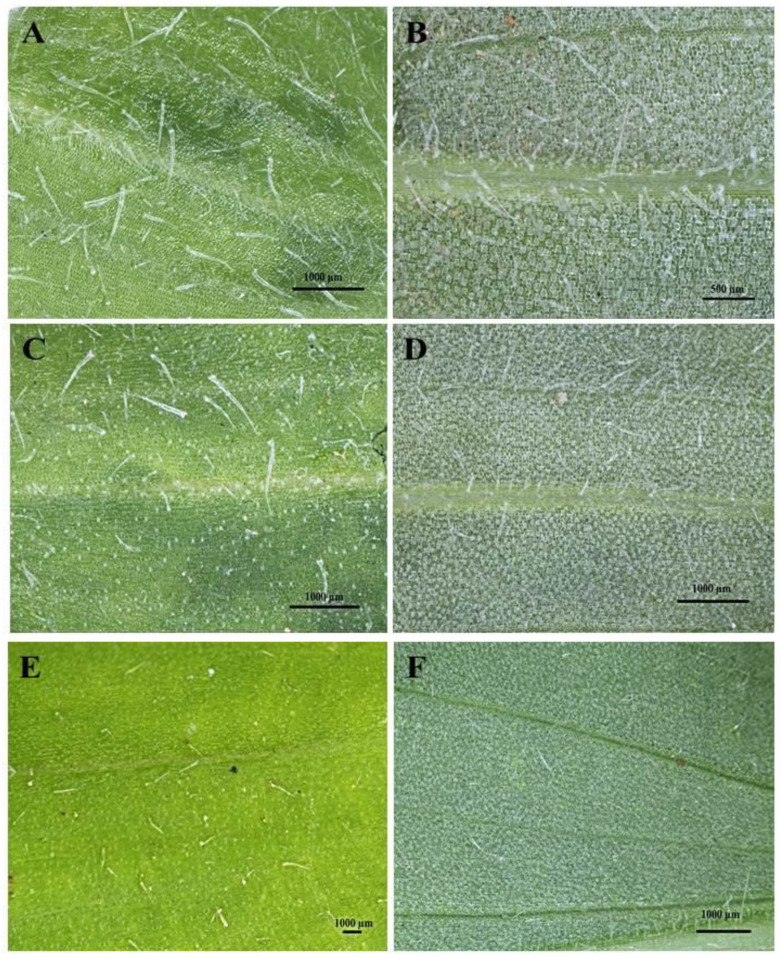
Stereomicrographs of non-glandular trichomes on the adaxial and abaxial surfaces of *C. benghalensis* leaves. (**A**) Adaxial surface of emergent leaf; (**B**) Abaxial surface of emergent leaf; (**C**) Adaxial surface of young leaf; (**D**) Abaxial surface of young leaf; (**E**) Adaxial surface of mature leaf; (**F**) Abaxial surface of a mature leaf.

**Figure 3:**
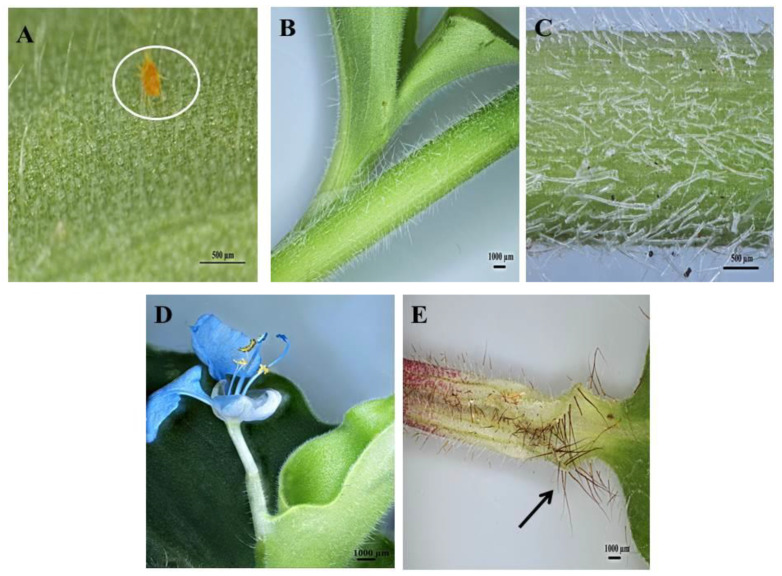
Stereomicrographs of non-glandular trichomes found on *C. benghalensis* plant parts. (**A**) Insect trapped among leaf trichomes; (**B**,**C**) Stem; (**D**) Flower and petiole; (**E**) Leaf sheath (red trichomes, arrow).

The developmental stages of *C. benghalensis* leaves can be seen in Figure 1. *C. benghalensis* carries NGTs on the adaxial and abaxial surfaces of the leaves, stem, petiole and leaf sheath (Figure 2A–F, Figure 3A–E). They are transparent except for those found on the leaf sheath. The trichome densities in the leaves and stem differ, with decreasing densities in the leaves due to leaf expansion. Trichomes densities appear to be relatively high on the adaxial and abaxial surfaces of emergent leaves (Figure 2A,B) in comparison to the mature leaves (Figure 2E,F). NGTs physically protect the plant against abiotic and biotic stresses [17,41]. With plant leaf expansion and growth, the trichomes become spaced further apart; however, if new trichomes are not produced on mature leaves, there will be an evident sparse indumentum [42]. Emergent leaves require increased protection as they are more susceptible to pathogen and insect attacks, possibly due to their elevated nutritional value [43,44,45].

Upon microscopic evaluation, the trichomes appear denser along the midrib of the abaxial leaf surface (Figure 2B,D,F). Studies have shown that the role of trichomes becomes less important as leaf development progressed. During this period, trichomes senesce and drop off [44]. Trichomes protect the plant against damage from biotic factors such as herbivores or ovipositing insects [46,47]. An aphid is seen attempting to move between the leaf pubescence of *C. benghalensis* in Figure 3A. NGTs appear to restrict insect movements and may cause further entrapment [48]. The stem indumentum showed a high density of NGTs (Figure 3B,C) as did the petiole (Figure 3D). Trichome density is developmentally regulated and may be controlled by plant hormones [49]. The leaf sheath is covered with red NGTs (Figure 1 and Figure 3E). Key characteristics of *C. benghalensis* were also previously described by Faden [50].

### 3.2. Scanning Electron Microscopy of Leaves and Stems of C. benghalensis

**Figure 4:**
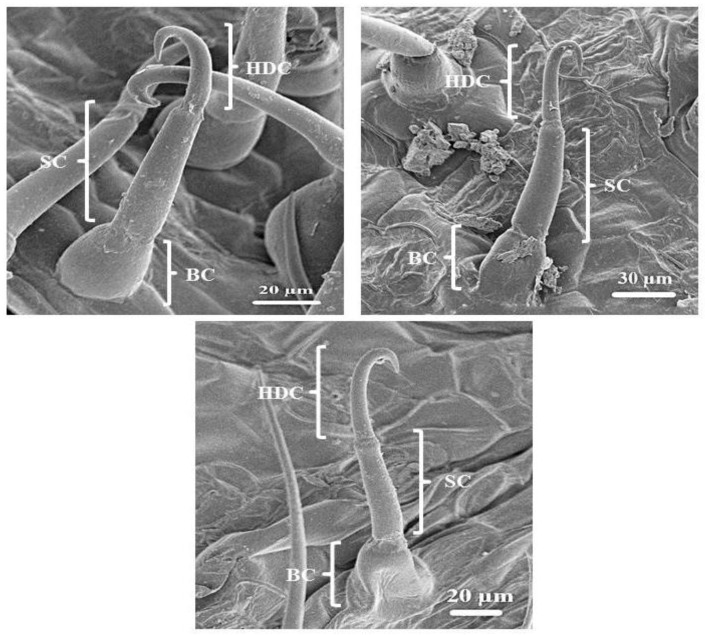
Scanning electron micrography of simple hooked non-glandular trichomes in all leaf stages and the stem of *C. benghalensis*. Abbreviations: BC: basal cell; SC: stalk cell; HDC: hooked distal cell.

**Figure 5:**
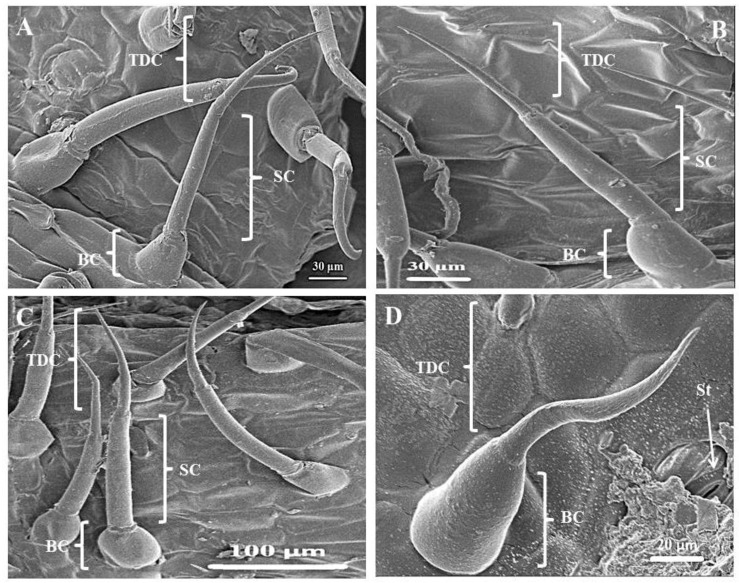
Simple bicellular and multicellular non-glandular trichomes on leaves and stems of *C. benghalensis*. (**A**) Multicellular NGT on the midrib; (**B**,**C**) Multicellular NGT; (**D**) Bicellular NGT on the leaf and stem. Abbreviations: BC: basal cell; SC: stalk cell; TDC: tapered distal cell; St: stomata.

The leaves and stems of *C. benghalensis* contain three morphologically distinct NGT types, bicellular, multicellular and multicellular hooked. The term “simple” is explained as trichomes being unicellular, uniseriate or unbranched [51]. The trichomes of *C. benghalensis* appear uniseriate and unbranched. The NGTs may be classified as simple bicellular (SB), simple multicellular (SM), or simple multicellular hooked (SMH) (Figure 4). The basal cell (Figure 4 and Figure 5) wedges into the epidermis, where it becomes thickened and almost bell-shaped. The uppermost cells of the SM (Figure 5A–C) and SB (Figure 5D) trichomes are tapered. The bicellular NGTs are less frequent than the hooked and multicellular trichomes. There was no evidence of glandular secreting trichomes (GST) on the leaves and stems of *C. benghalensis.* Non-glandular hooked and multicellular trichomes consisted of three cells, including the basal cell. The cell walls of each NGT type are of varying thickness. Similar results were found in a study that looked at the epidermal features of *C. benghalensis* [52,53].

The anatomical features of Commelinaceae were classified by Tomlinson [54] and it was proposed that apart from the GST, NGTs found in *Commelina* are two-celled, prickled, uniseriate with differing cell numbers and hooked. High densities of hooked NGTs were also found on the spathe and leaves of *Commelina erecta* [55]. NGTs act as a physical barrier against various external factors such as insects and animals [17]. They protect against ultraviolet radiation, extreme temperatures, and water loss [17,56]. The NGT’s spine-like and hooked nature allows for direct impaling of an insect’s body hindering insect feeding behavior [47].

### 3.3. Survey Sections of Leaves and Stem of C. benghalensis Embedded in Resin

**Figure 6:**
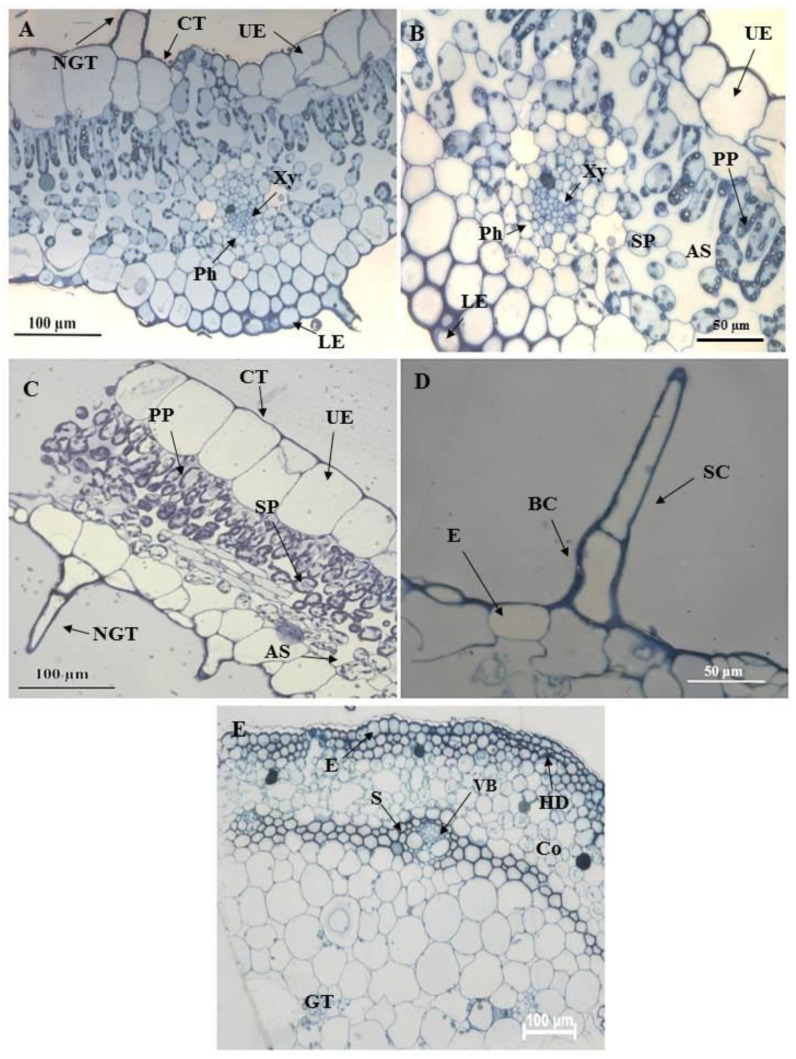
Light micrographs of resin embedded leaf and stem sections of *C. benghalensis*. (**A**,**B**) The midrib of an emergent leaf; (**C**) Segment of a leaf with a non-glandular trichome; (**D**) Non-glandular trichome on the epidermal layer of a leaf section; (**E**) Stem section Abbreviation: CT: cuticle; UE: upper epidermis; LE: lower epidermis; PP: palisade parenchyma; Xy: xylem; Ph: phloem; SP: spongy parenchyma; NGT: non-glandular trichome; BC: basal cell; SC: stalk cell; VB: vascular bundle; E: epidermis; HD: hypodermis; S: sclerenchyma; Co: collenchyma; GT: ground tissue; AS: air space.

The transverse section of the leaf midrib consisted of an upper and lower epidermis, xylem, and phloem (Figure 6A). A cuticle covers the epidermis. The cells of the epidermis are polygonal in shape and conform to a straight, anticlinal wall pattern. There are numerous air spaces between the spongy parenchyma cells (Figure 6B,C). The vascular bundle appeared to be closed (Figure 6B). NGTs were present on the upper and lower epidermis (Figure 6A,C). The stem section has a circular outline (Figure 6E). The stem consists of the epidermis, hypodermis, vascular bundle, and ground tissue. Previous studies had shown similar results [53,57].

### 3.4. Ultrastructural Analysis Using Transmission Electron Microscopy

**Figure 7:**
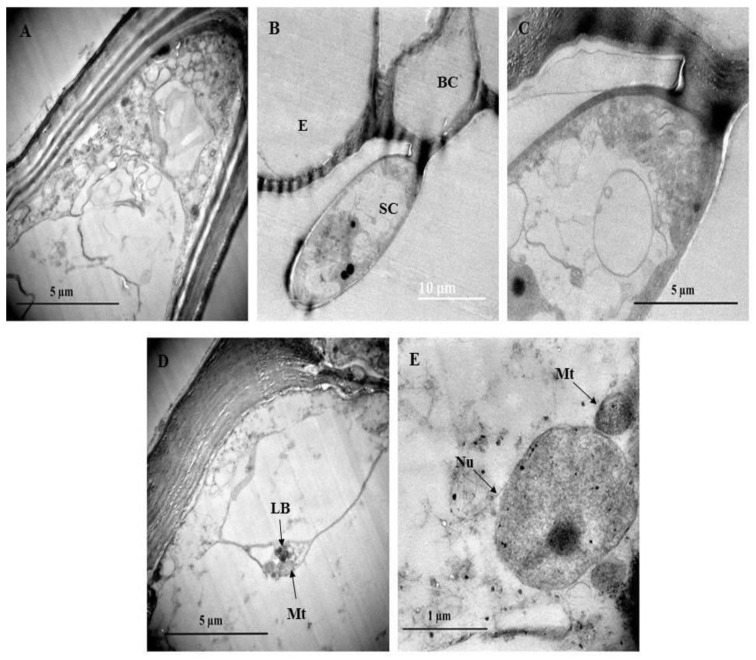
Transmission electron micrograph of non-glandular trichomes found on *C. benghalensis* leaves. (**A**,**B**) Non-glandular trichomes on the leaf epidermis; (**C**) Cytoplasm containing electron dense material within the trichome; (**D**) Mitochondria and lamellar bodies located toward the periphery of the cell; (**E**) Nucleus and mitochondria within the cytoplasm. Abbreviations: BC: basal cell; SC: stalk cell; E: epidermis; LB: lamellar bodies; Mt: mitochondria; Nu: nucleus.

Transmission electron micrographs showed various metabolically active organelles within the trichome (Figure 7). The cell wall of the trichomes appears to be highly cutinized (Figure 7A) especially between the stalk and basal cell of the trichome (Figure 7B,C). Lamellar bodies and mitochondria can be seen toward the periphery of the cell (Figure 7D). Lamellar bodies are specialized structures for storing and secreting certain lipids [58]. Figure 7E shows an enlarged nucleus adjacent to a mitochondrion. The mitochondria aid in adenosine triphosphate (ATP) generation that drives the cell’s fundamental functioning [59].

**Figure 8:**
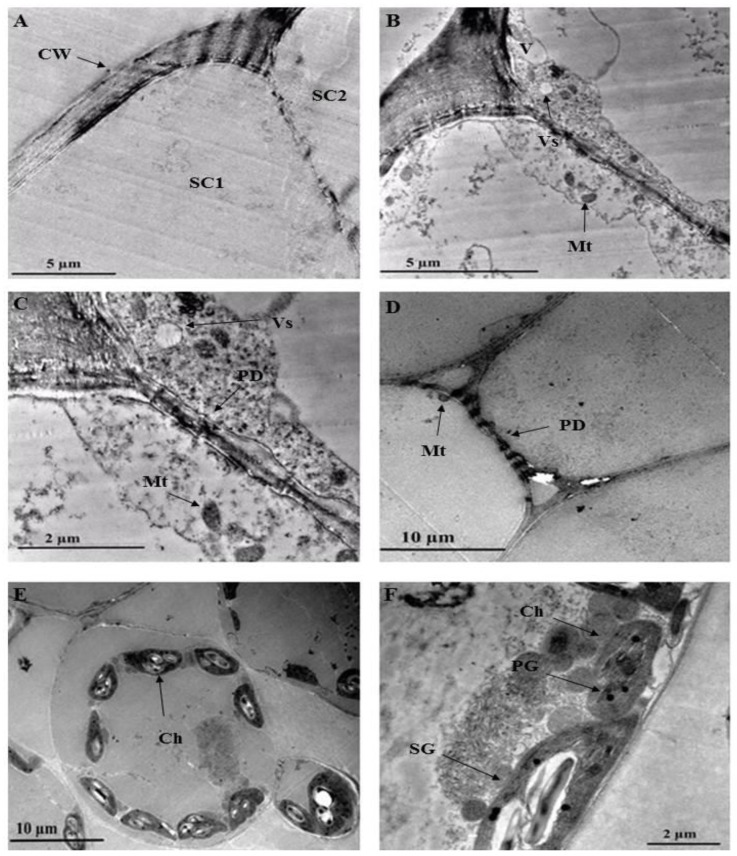
Transmission electron micrographs of *C. benghalensis* leaf section. (**A**–**C**) Trichome; (**D**–**F**) Leaf tissue containing chloroplasts and starch grains. Abbreviation: CW: cell wall; SC1: stalk cell 1; SC2: stalk cell 2; V: vacuole; Vs: vesicle; Mt: mitochondria; PD: plasmodesmata; Ch: chloroplast; PG: plastoglobuli; SG: starch grain.

Organelles such as vesicles and vacuoles are shown in Figure 8B. With the vesicle in close proximity to the plasma membrane, it could be speculated that larger molecules such as polysaccharides and proteins or other secretory substances are being transported [41,60,61]. Plasmodesmata are observed in the trichome and leaf cell (Figure 8C,D). Plasmodesmata between the trichome stalk cells act as cytoplasmic channels that aid in plant cell connectivity, through which nutrients and growth and development signals are transferred [60]. The presence of plasmodesmata suggests that there could be intercellular transport between the NGT and leaf. Figure 8C,D also show a mitochondrion close to the plasma membrane. Mitochondria can be located along membrane surfaces when a cell’s plasma membrane is highly active in the transportation of compounds in or out of the cell [61]. Numerous chloroplasts are evident in the leaf section (Figure 8E). These chloroplasts contain plastoglobuli and starch grains (Figure 8F).

Multiple starch grains within the chloroplast may act as storage products and accumulate only during active photosynthesis is taking place [61]. Plastoglobuli are lipoprotein globules found in plastids and function as a lipid reservoir, mediate the plant stress response, disassemble the thylakoid in senescing tissues, and act in the transition of chloroplast to chromoplast [62].

### 3.5. Localisation of Bioactive Compounds within Trichomes of C. benghalensis

**Figure 9:**
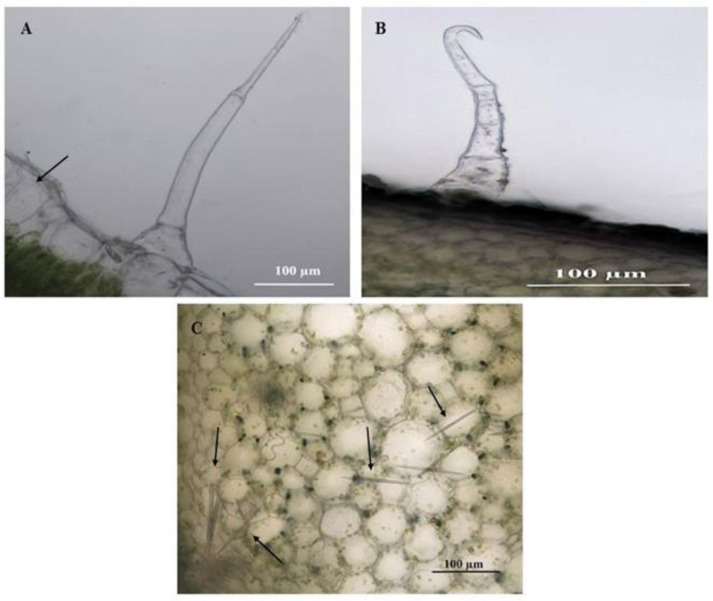
Unstained leaf and stem sections of *C. benghalensis*. (**A**) Multicellular non-glandular trichome on a leaf that contains raphide crystals; (**B**) Hooked non-glandular trichome on the stem; (**C**) Raphide crystals inside stem tissue (Arrow).

SM and SMH NGTs are distinct in the unstained leaf and stem sections of *C. benghalensis* ((Figure 9A,B). Raphide crystals are present within the stem and epidermal layer of the leaf section (Figure 9A,C). The needle-like raphide crystals are larger in size in the stem section as compared to their minute size in the leaf section. These crystals appear needle-shaped and appear singularly or in clusters. The raphide crystals are made of calcium oxalate (CaOx) [63]. These CaOx crystals usually occur in meristematic tissues in plants where the transport of organic molecules is regulated by calcium ions and is a form of metabolic waste [64]. The functions of CaOx in plants are to regulate calcium levels in tissues and organs, detoxify heavy metals and prevent herbivory [65]. Previous studies have reported similar needle-like raphide crystals that were found in *C. benghalensis* leaves and stems [57,66].

**Figure 10:**
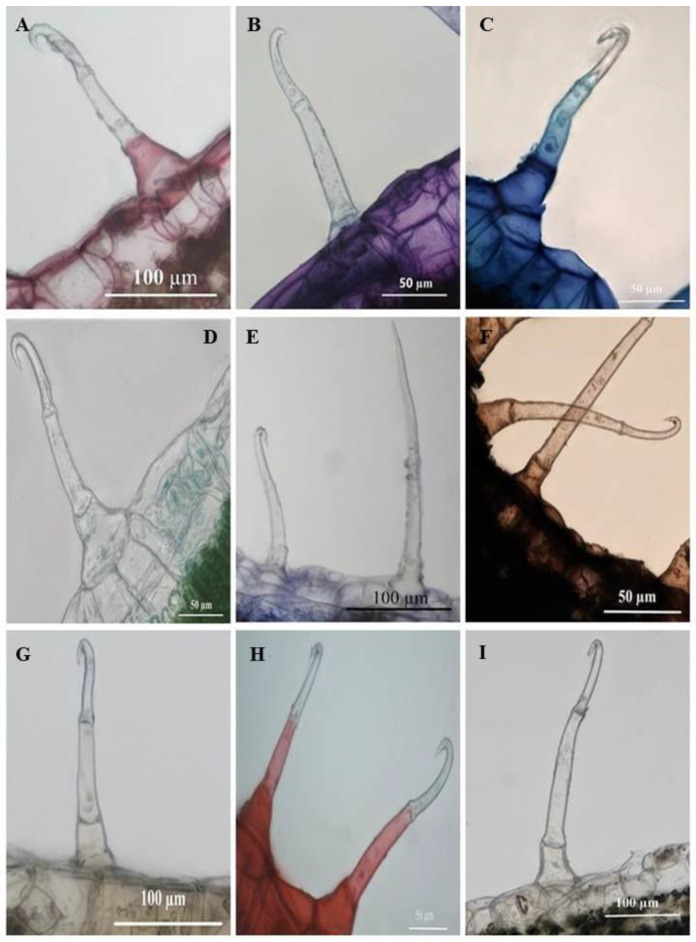
Transverse sections of histochemically stained leaf sections of *C. benghalensis*. (**A**) Mucilage and pectin present in basal cells (Ruthenium red); (**B**) Toluidine blue for carboxylated polysaccharides (purple) and polyaromatic substances (blue); (**C**) Basal and stalk cell nucleoproteins (Methylene blue); (**D**) Cellulosic cell walls present in the basal cell (Fast green); (**E**) Coomassie blue stained proteins in the basal cell; (**F**) Lipids accumulated throughout the trichome (Sudan black); (**G**) Ferric chloride stained phenols in the basal and stalk cells (orange-brown); (**H**) Lignified cell walls in the basal and stalk cells stained red (Safranin); (**I**) Alkaloids were present in the basal and stalk cells stained with Wagner’s and Dittmar reagent (brown-orange).

**Figure 11:**
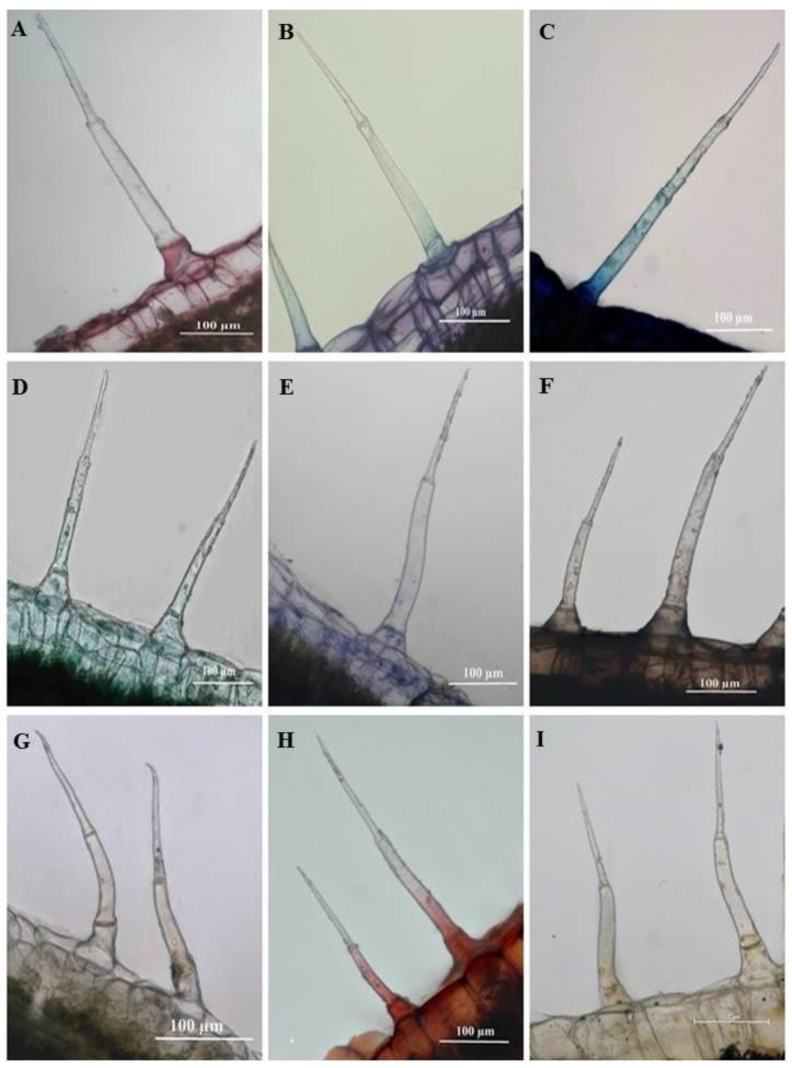
Light micrographs of histochemically stained leaf sections of *C. benghalensis*. (**A**) Ruthenium Red stained basal cell red (mucilage and pectin); (**B**) Toluidine blue stained polyaromatic substances (blue) and carboxylated polysaccharides (purple); (**C**) Basal and stalk cell indicating nucleoproteins (Methylene blue); (**D**) Cellulosic cell walls in the basal cell indicated by Fast green; (**E**) Coomassie blue stained proteins in the basal cell; (**F**) Lipids accumulated throughout trichome (Sudan black); (**G**) Ferric chloride stained the basal and stalk cells orange-brown stain (phenols); (**H**) Lignified cell walls in the basal and stalk cells stained red (Safranin); (**I**) Alkaloids were present in the basal and stalk cells stained with Wagner’s and Dittmar reagent (brown-orange).

Histochemical analyses highlighted the locality of several important phytochemical compounds in plant tissues and trichomes. Ruthenium red had stained the basal cells of the SMH (Figure 10A) and SM trichomes (Figure 11A) a pinkish-red, a positive indication of mucilage and pectin in the cells. Sections stained with Toluidine blue reveal carboxylated polysaccharides (purple) in the tissue while the basal cells of the trichomes (Figure 10B and Figure 11B) stained a light blue signifying the presence of polyaromatic substances. The basal and stalk cells of the SMH (Figure 10C) and SM (Figure 11C) trichomes stained with Methylene blue exhibited nucleoproteins. Sections stained with Fast Green were light green in the basal cells of the SMH (Figure 10D) and SM (Figure 11D) trichomes highlighting cellulosic cell walls. Figure 10E and Figure 11E showed proteins in the basal cell of SMH and SM, respectively, stained with Coomassie blue.

Sudan black was used to identify lipids in plant tissues and trichomes staining blue to black [34]. As shown in Figure 10F and Figure 11F, the entire SMH and SM trichome stained dark brown, indicating the presence of lipids. Phenols were present in the stalk and basal cells of SMH (Figure 10G) and SM (Figure 11G) trichomes as indicated by the brown deposits brought upon by Ferric chloride. Safranin stained the stalk and basal cells of SMH (Figure 10H) and SM (Figure 11H) red revealing lignified cell walls. Alkaloids were present in the basal and stalk cells of the SHM (Figure 10I) and SM (Figure 11I) trichomes indicated by the orange-brown stain.

Several bioactive compounds found in trichomes aid in the host’s defense and overall physiology [19]. Phenolic compounds can polymerize to form a glue that sticks insects to the leaf surface [67,68]. Due to the selection pressure of competing herbivores, alkaloids in plants have evolved to form chemical defenses against herbivores and pests [69]. Lignin is responsible for the plant organ’s mechanical support being cell wall polymers, defense against pathogens and herbivores and water transport through xylem vessels [70,71,72]. Proteins such as proteinase inhibitors may accumulate in plant tissues once the tissue is wounded [18] to inhibit an insect or animal’s digestive proteins once the plant is eaten, interfering with the herbivore’s physiology [18].

**Figure 12:**
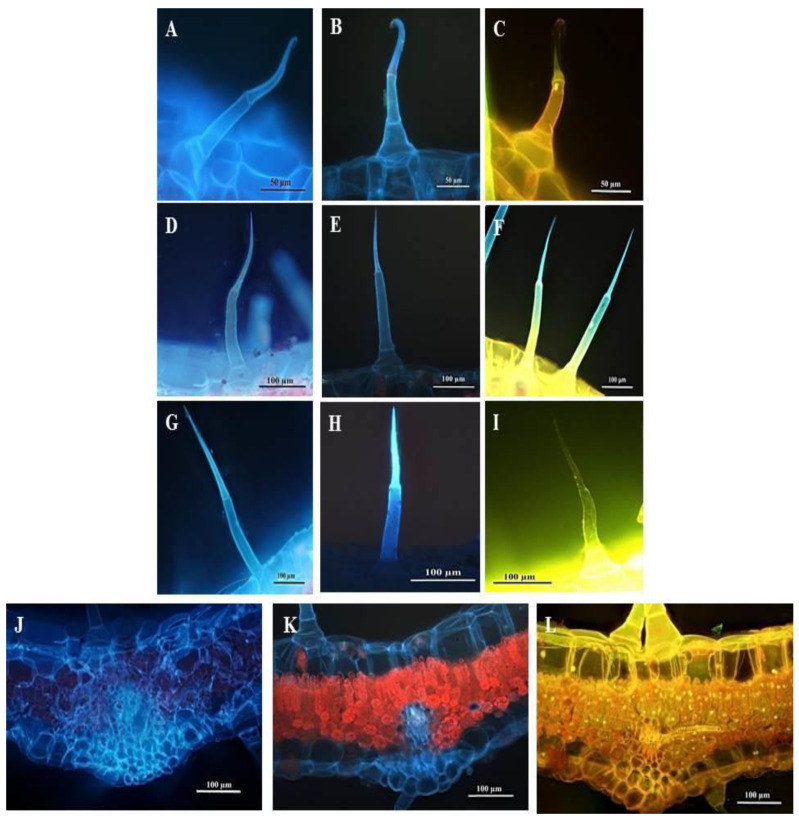
Fluorescent micrographs of leaf and stem sections of *C. benghalensis*. (**A**,**D**,**G**,**J**) Sections stained with Calcofluor white (cellulose); (**B**,**E**,**H**,**K**) Autofluorescence of sections (phenols); (**C**,**F**,**I**,**L**) Sections stained with Acridine orange (cell viability); (**A**–**C**) Hooked trichomes on leaf sections; (**D**–**F**) Multicellular trichomes on leaf sections; (**G**–**I**) Multicellular trichomes on stem sections; (**J**–**L**) Stained leaf sections.

Fluorescence micrographs showed that the leaf section and trichomes present on the leaves and stems stained with Calcofluor white fluoresced bright blue, indicating cellulose in the cell walls (Figure 12A,D,G,J). The blue autofluorescence of the leaf section and leaf and stem trichomes illustrated the presence of phenols (Figure 12B,E,H,K). Leaf sections and trichomes stained with Acridine orange fluoresced bright yellow-green-blue, indicating cell and trichome viability (Figure 12C,F,I,L).

NGTs are not known to synthesize, accumulate or liberate bioactive compounds [17]. There is, however, little evidence to suggest that NGTs are incapable of low levels of secretion [19]. The histochemical analyses performed on the NGTs of *C. benghalensis* showed the ability to accumulate various phytochemicals. Similarly, Tozin et al. [41] performed histochemical analyses on NGTs of Lamiaceae and Verbenaceae which showed that the NGTs synthesize and accumulate biologically active compounds. An accumulation of secretions was found in the apex of NGTs of *Lantana camara* [41]. Cells within NGTs are metabolically active during early development and may remain active at maturity [41,61,73]. Acridine orange stain showed that cells in the SMH and SM trichomes of *C. benghalensis* are viable.

Previous phytochemical studies on *C. benghalensis* whole plant, leaves and/or stems showed the presence of phytocompounds such as carbohydrates, polyphenols, alkaloids, resins, flavonoids, proteins, phlabotannins, volatile oils, terpenoids, saponins, balsams, caffeine, glycosides, tannins, gum and mucilage [5,10,13,74,75,76,77,78,79,80,81,82,83]. One speculation for the accumulation of metabolites in the NGTs could be that it forms part of a plant’s chemical defense against herbivores, insects or pathogens [45]. These bioactive compound in the leaves and stems could possibly be translocated from the plant cell into the trichome cell by means of simple diffusion, symplastic or apoplastic transport, vesicle mediated movement or transporter-mediated membrane transport [84,85]. This could be theorized as one possible explanation for the presence of lamellar bodies and mitochondria within the NGT cell as well as the vesicle in close proximity to the plasma membrane of the plant cell. However, further research needs to be performed for greater detail.

## 4. Conclusions

Histochemical analysis revealed various pharmacologically important bioactive compounds, such as phenolics, alkaloids and cellulose, within the NGTs. These results show that the NGTs are metabolically active as they contain organelles such as vacuoles and mitochondria and can retain and possibly synthesize various bioactive compounds, expressing a characteristic similar to glandular trichomes. It can be speculated that the movements of substances such as metabolites or large molecules are transported by vesicles located within the trichomes. The presence of the mitochondrion near the plasma membrane suggested that the membrane is highly active with the movement of substances in and out of the cell. The subcellular contents of the trichomes were not previously investigated. By understanding the contents of the NGTs in *C. benghalensis* on a cellular and biochemical level, the production of medicinally significant metabolites within the trichome can be strengthened and augmented in the future by means of plant biotechnology [45,68] as access to these epidermal extremities is relatively easy.

Detailed compound isolation studies should be conducted solely on the trichomes as histochemistry displayed some evidence of the presence of secondary metabolites within the trichomes of *C. benghalensis*. Further research should identify the stomatal and trichome density and length on the adaxial and abaxial surfaces over the leaf developmental stages, stem, floral organs, and root, as well as investigating the chemical constituents of trichomes over all the developmental stages. New research will add to the existing body of knowledge for this species’ trichomes and enable possible identification of its growth patterns, function/s, and significance to the plant.

## Data Availability

Not applicable.
